# Association between helminth infection and allergic disorders among children in Batu, Ethiopia

**DOI:** 10.1002/iid3.1222

**Published:** 2024-03-22

**Authors:** Sosina Walelign, Mheret Tesfaye, Geremew Tasew, Kassu Desta, Aster Tsegaye, Bineyam Taye

**Affiliations:** ^1^ Department of Medical Laboratory Sciences Addis Ababa University Addis Ababa Ethiopia; ^2^ Bacteriology and Mycology National Reference Laboratory Ethiopian Public Health Institute Addis Ababa Ethiopia; ^3^ Bacterial, Parasitic, and Zoonotic Diseases Research Directorate Ethiopian Public Health Institute Addis Ababa Ethiopia; ^4^ Department of Biology Colgate University Hamilton New York USA

**Keywords:** allergy, atopy, helminths, IgE

## Abstract

**Background:**

Helminths are potent immunomodulators and in their chronic infection state they may protect against allergy‐related disease and atopy. However, they are also known for inducing allergic conditions. This study aimed to assess the association between helminths,  atopy and allergic conditions.

**Methods:**

A total of 461 school children participated in this cross‐sectional study. Data on allergic symptoms and a range of confounding variables was gathered from parents via an interviewer‐led questionnaire. Skin sensitization to house dust mite and cockroaches was analyzed, and a stool sample was collected for helminth analysis. Serum total Immunoglobulin E using enzyme‐linked immunosorbent assay and eosinophil count were also measured.

**Results:**

Overall sensitivity to both allergens was 2.4%. Self‐reported allergic outcomes in the last 12 months for the 461 participants had been : wheezing 3.7%, asthma 2.2%, eczema 13.2% and hay fever 6.9%. Overall, the prevalence of helminth infection was 11.9% (53/444). A borderline significant association was found between atopy and any allergy symptoms (odds ratio [OR]: 3.32, 95% confidence interval [95% CI: 0.99, 11.1], *p* = .052). There was no significant association between helminths and atopy (OR: 0.64 [95% CI: 0.29, 1.41], *p* = .268) and also between helminths and allergic symptoms (OR: 0.64 [95% CI: 0.29, 1.41], *p* = .268). Bivariate analysis showed keeping an animal in the house increases the risk of atopy while maternal and paternal history of allergy increases the risk of developing allergic symptoms in the children.

**Conclusion and clinical relevance:**

This study found a non‐significant inverse association between helminths infection and atopy and allergic disorders, likely due to reduced statistical power, resulting in a lower prevalence of atopy and allergic conditions. A high powered longtitudinal study is necessary to explore the casuality and potential therapeutic benefits of helminths for allergic disorders.

## Graphical abstract

A cross‐sectional study in Batu, Central Ethiopia involving 461 school children, to determine the association between helminths and allergy/atopy. Even though the analysis shows a nonsignificant association, the number of helminth‐infected participants have a noticeable low atopic and allergic conditions, indicating an inverse association.

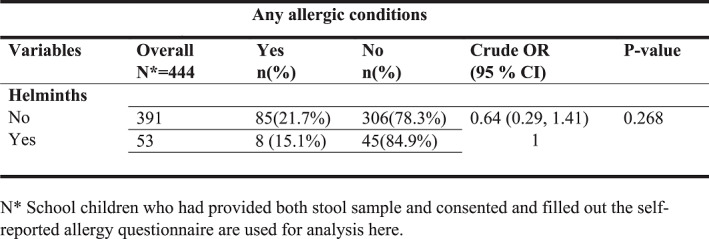

## 1 INTRODUCTION

Helminth infections and allergy disorders are common causes of morbidity around the globe, although their distribution shows an inverse association.^1^, ^2^, ^3^ A potential role of parasitic infections having a protective role against autoimmune disorders by parasite‐mediated immune suppression was implicated in the 1960s by Greenwood.^4^

The human immune response to a geohelminth infection and allergic disease resemble each other. Helminth infections have been associated with a regulatory state that impairs both responses to parasite specific and bystander antigens. Some immune regulatory mechanisms helminths use are regulatory T‐cell expansion,^5^ enhanced production of interleukin‐10, transforming growth factor‐β,^6^ and M2 macrophages.^7^

The interest that rose in the 1960s to investigate the effect of helminths on allergy still continues today without reaching a consensus on their protective or inducing role. Regardless of the conflicting results from studies, the current science has shown advancement in working on trials that aim to treat immune‐mediated diseases using helminth‐derived molecules. Such helminth‐derived molecules could be used to selectively mimic the desirable regulatory effects of parasitic helminths, without the side‐effects of infection, in new therapeutic approaches for immune‐mediated diseases.^8^, ^9^, ^10^, ^11^

Low‐income countries are highly burdened with intestinal helminth infections,^12^ making them an ideal place to conduct studies on the interaction between atopic disorders and intestinal helminth infections. On the other hand, there is an increase in allergy^3^ in some urban areas of developing countries, which might be linked to improved treatment in urban rather than rural areas. Hence, understanding the relationship between helminths and allergy could gear future treatment strategies and provide insight into the risks and benefits of eradicating helminth infections in endemic areas. Therefore, we studied the association between helminth infections and atopy or allergic diseases using Ethiopian children.

## 2 METHOD

### 2.1 Study population and design

The study was conducted in Batu town,formerly known as Ziway, located 160 km south of Addis Ababa, the capital of Ethiopia. Batu has a population of 43,660 as per the 2007 census. A cross‐sectional study was designed and data was collected from three locations: Sher Elementary School, Sher Ethiopia Hospital, and Batu Hospital. A convenient sampling technique was used to recruit eligible participants: school children <15 years old, who lived in Batu town during the study period (May–June 2016).

### 2.2 Data collection

Written informed consent was obtained from parents before delivering an interviewer‐led questionnaire. Data on allergic disease symptoms and various potential confounders (e.g., environmental factors, asthma and allergy history in the family, socio‐demographic characteristics, and so on) were obtained from the questionnaire. Atopic status was determined by skin prick test (SPT) using two allergens; house dust mite (*Dermatophagoides pteronyssinus)* and German cockroach (*Blattella germanica*) allergens (Inmunotek inc. immunologies, Germany). Glycerol saline and histamine dihydrochloride were used as negative and positive controls, respectively. A positive test was defined as an average of two perpendicular wheal diameters, one of which was the maximum measurable diameter, of at least 3 mm greater than the saline control response.

Stool and blood samples were collected on site and transported to local laboratories, the Ethiopian Public Health Institute (EPHI) and the Department of Medical Laboratory Sciences of AAU, Addis Ababa, for further analysis.

### 2.3 Laboratory analyses

ELISA for serum total IgE (Diagnostic Automation, Cortez Diagnostics Inc.) was carried out at EPHI. This IgE quantitative test is a solid phase enzyme‐linked immunosorbent assay based on the sandwich principle.^13^

Stool examination for parasite detection was done using the wet mount and formol ether concentration technique. the latter technique used an applicator to homogenize about 1 g of the stool sample in 8 mL of sodium acetate‐SAF. The resulting emulsification was sieved before collection in 4 mL of diethyl ether. The tube containing these contents was capped with a rubber stopper before 30 s of inversion, followed by centrifugation for 1 min at 3000 rpm. After decanting off the supernatant (top three layers), the remaining sediment was subjected to microscopic examination for parasitic ova and larvae^14^ and manual differential eosinophil counts using an automated hematological analyzer (Mindray BC‐3000 Plus auto hematology analyzer).^15^ Manual differential count A Wright stained blood film was systematically examined for the different white cells seen in each field, until a total of 100 cells were counted. The absolute number of each white cell type was calculated by multiplying the number of each cell counted (expressed as a decimal fraction) by the total white blood cell count obtained from the complete blood cell count from the hematology analyzer.^14^

### 2.4 Statistical analysis

Data was entered into IBM SPSS version 20 (SPSS INC) for analysis. Data consistency was checked by tabulating variables and making simple frequencies using SPSS. Descriptive statistics were used to summarize continuous variables while simple frequencies were used to show the distribution of the socio‐demographic and clinical characteristics of the participants. Crosstabs and binary logistic regression tests were used to establish an association between categorical variables. The Kruskal–Wallis test was used to compare the means of serum total IgE and the Independent sample median test was used to check if there was a significant difference in eosinophil distribution among the different participant groups. Bivariate analysis was done to calculate the unadjusted odds ratio/adjusted odds ratio with 95% confidence interval (95% CI) to quantify the strength of association between atopy/allergy symptoms and helminth infections and other possible predictors of atopy/allergy symptoms. A *p* value less than 0.05 was considered statistically significant.

### 2.5 Ethical consideration

Ethical clearance was obtained from the departmental research and ethics review committee (DRERC/197/16/MLS) of the Department of Medical Laboratory Science. Permission was obtained from the hospitals, health centers and school authorities to conduct the study. Written informed consent (signed or thumb print) and assent were gained from each participant and their guardians. Confidential identifiers were used to code participant's identities. Participants with parasites were provided with the appropriate anti‐helminth medication as per the local guideline. Results and any information regarding patients are kept confidential during and after the completion of the research project by password‐protected electronic programs and locking hard copy files.

## 3 RESULT

### 3.1 Demographic and socioeconomic characteristics of study participants

A total of 461 eligible study participants were recruited and just over half were female (50.8%) and the majority (96.1%) were from urban areas. The mean age was 8.8 years (the age range being between 2 and 14 years). Maternal demographic characteristics showed 57.9% of the participants’ mothers had a formal education and 43.8% were housewives.

### 3.2 Allergic sensitization and self‐reported allergic symptoms

Skin Prick Test was performed for 454 participants for the two allergens, house dust mites (*D. pteronyssinus)* and German cockroach (*B. germanica*), with rate of 1.1% and 1.5% sensitivity, respectively. Overall sensitivity or atopic status was 2.4%. Only one participant was sensitive to both allergens. Self‐reported allergic outcomes in the last 12 months for the 461 participants had : wheezing (3.7%), asthma (2.2%), eczema (13.2%), and hay fever (6.9%) (Table 1).

**Table 1:** Frequency of allergic sensitizations and self‐reported allergic symptoms, Batu, Ethiopia 2016.

Variables	Number	%
Self‐reported allergic symptoms (*N* = 461)	
Wheezing	17	3.7
Asthma	10	2.2
Hay fever	32	6.9
Eczema	61	13.2
Any allergy symptom	94	20.4
Skin prick test,SPT (*N* = 454)		
HDM (*Dermatophagoides pteronyssinus)*	5	1.1
German cockroach (*Blattella germanica*)	7	1.5
Sensitization to both allergens	1	0.22
Any sensitization^a^	11	2.4
Both atopic and allergic	5	1.1

Abbreviation: HDM, house dust mite.

^a^ Skin sensitization (atopic status) either to *D. pteronyssinus* or German cockroach (*B. germanica*) or to both allergens.

### 3.3 Distribution of helminth parasites

Overall, the prevalence of any helminth infection was 11.9% (53/444). *Hymenolepis nana* was found to be the most frequent helminth, followed byEntrobius Vermicularis, Schistosoma mansoni, Tenia species, hookworm species, *Ascaris lumbricoides and Tricuris trichuria*.

### 3.4 Association of helminth infections with allergic symptoms and atopy

Since the prevalence of individual helminths was low, the analysis was done using oveall helminth data. Similarliry, overall allergic symptoms and skin sensitization data were used. However, Hay fever (6.9%) and eczema (13.2%) were individually analyzed to determine their association with helminth infection. However,the analysis showed that there was no significant association between them. Our result showed a non‐significant inverse association between helminth infections and atopic and allergic disorders. OR: 0.64 [95% CI: 0.29, 1.41], *p* = 0.268) (Table 2) OR: 0.52 [95% CI: 0.11, 2.51], *p* = 0 .413) (Table 3), respectively.

**Table 2:** Association between allergic conditions and helminth infections, Batu, Ethiopia, 2016.

	Any allergic conditions				
Variables	Overall *N* ^a^ = 444	Yes *n* (%)	No *n* (%)	Crude OR (95% CI)	*p*
Helminths					
No	391	85 (21.7%)	306 (78.3%)	0.64 (0.29, 1.41)	.268
Yes	53	8 (15.1%)	45 (84.9%)	1	

Abbreviations: CI, confidence interval; OR, odds ratio.

^a^ School children who had provided both stool sample and consented and filled out the self‐reported allergy questionnaire are used for analysis here.

**Table 3:** Association between atopy and helminth infection among school children of five selected facilities, Batu, Ethiopia, 2016.

	Atopy (skin sensitization)				
Variables	Overall *N* a = 438	Yes *n* (%)	No *n* (%)	Crude OR (95% CI)	*p*
Helminths					
Yes	387	8 (2.1%)	379 (97.9%)	0.52 (0.11, 2.51)	.413
No	51	2 (3.9%)	49 (96.1%)	1	

Abbreviations: CI, confidence interval; OR, odds ratio.

^a^ School children who had provided both stool sample and were willing to test for skin prick test.

### 3.5 Total IgE and eosinophil levels among the different groups of study participants

**Figure 1:**
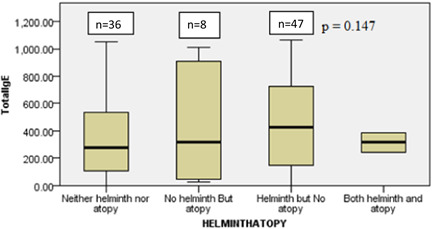
Distribution of total IgE among the different helminth/atopy groups Batu, Ethiopia, 2016.

**Figure 2:**
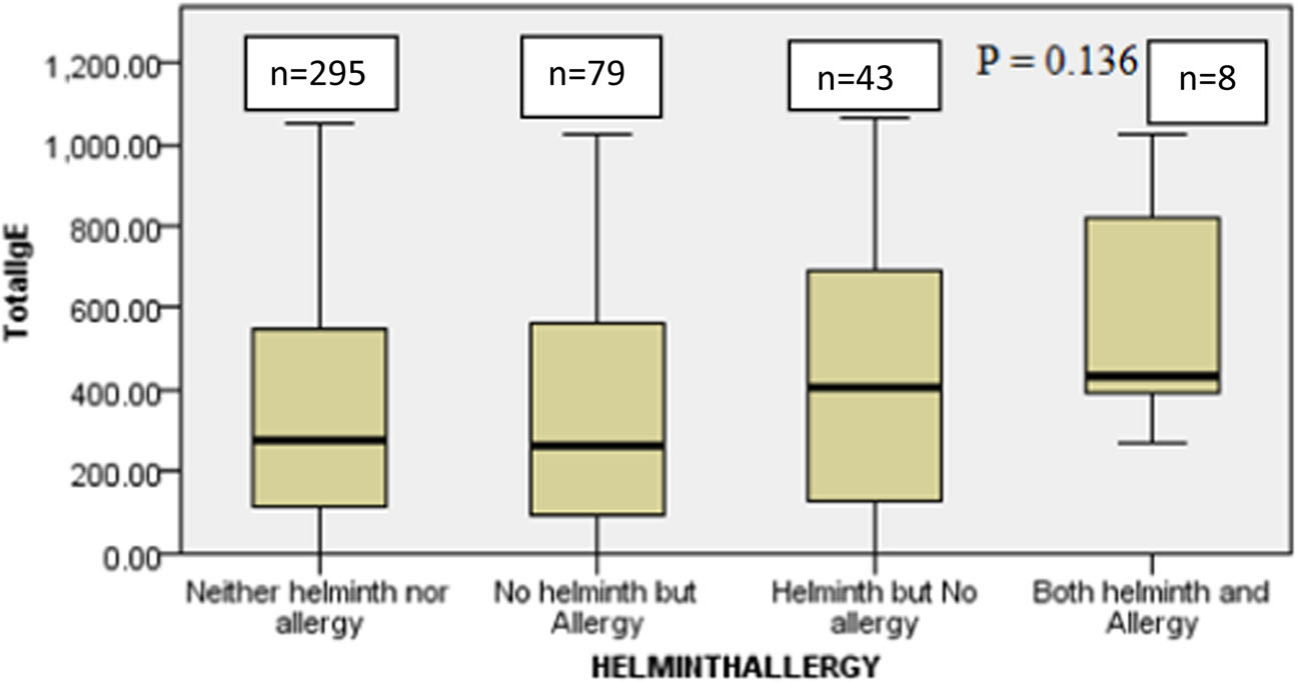
Distribution of total IgE among the different helminth/allergy groups Batu, Ethiopia, 2016.

Independent sample Kruskal–Wallis test was used to check if there was a significant mean rank difference of total IgE concentration distribution among the different helminth/atopy (Figure 1) and helminth/allergy (Figure 2) groups of the study participants. The analysis showed the distribution of total IgE did not differ significantly across the categories of both helminth/allergy (*p* = .136) and helminth/atopy (*p* = .147) groups. There is an increasing pattern in the total IgE level from “no helminths and no atopy” groups to those with “both helminths and atopy” groups (Figure 2).

**Figure 3:**
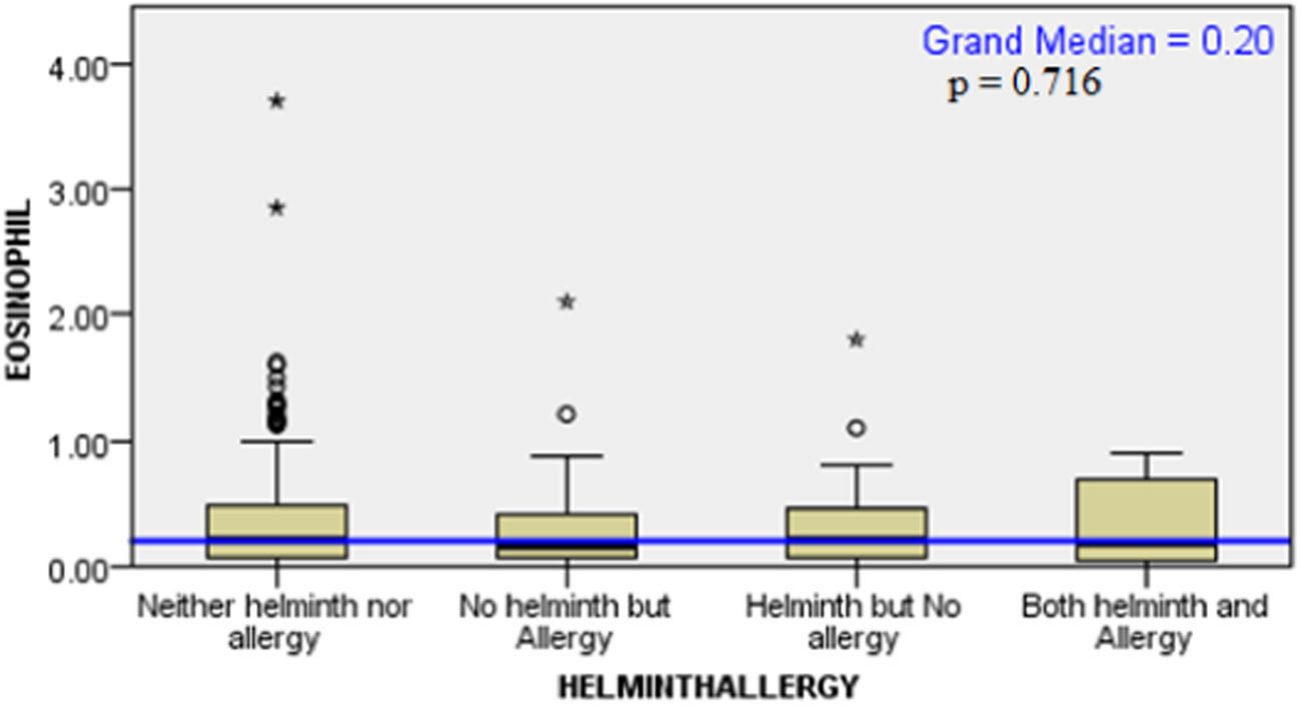
Distribution of eosinophil among the different helminth/allergy groups Batu, Ethiopia, 2016.

**Figure 4:**
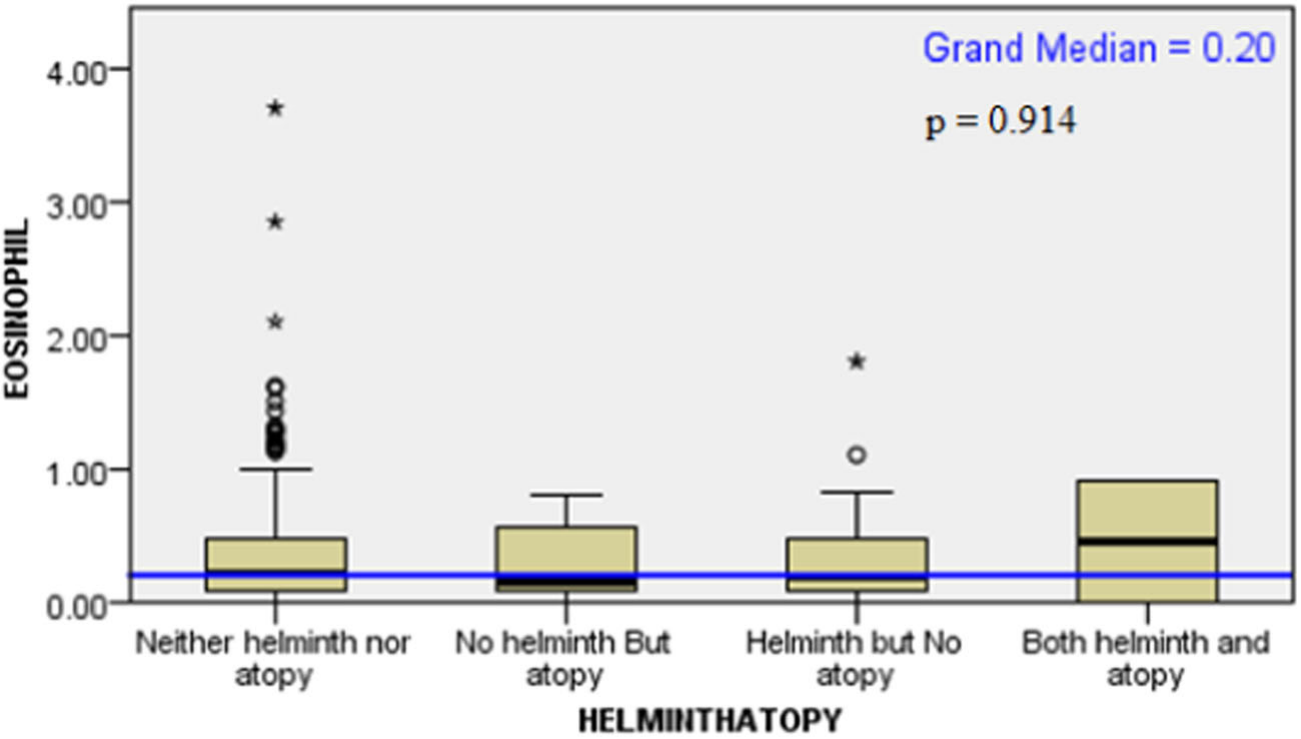
Distribution of eosinophil among the different helminth/atopy groups Batu, Ethiopia, 2016.

As there were outliers in eosinophil distribution, independent sample median test was used to check if there was a significant median difference of among the different helminth/allergy (Figure 3) and helminth/atopy groups (Figure 4). Both revealed a nonsignificant difference among the groups.

### 3.6 Association between skin sensitization (atopy) and allergy symptoms

A borderline significant association was found between atopy and any allergy symptoms. Individuals with a positive SPT response to any allergen were 3.32 times more likely to report any allergy symptom (OR 3.32 [95% CI: 0.99, 11.1], *p* = .052) (Table 4).

**Table 4:** Association between atopy and allergy symptoms, Batu, Ethiopia 2016.

	Allergy symptoms					
Variable	Overall *N*	Yes *n* (%)	No *n* (%)		Crude OR (95% CI)	*p*
Atopy					
Yes	11	5 (45.5%)	6 (54.5%)		3.32 (0.99, 11.1)	.052
No	443	89 (20.1%)	354 (79.9%)		1	

Abbreviations: CI, confidence interval; OR, odds ratio.

### 3.7 Potential risk factors for allergy and skin sensitization

Univariate analyses were conducted to investigate the impact of demographic factors and potential confounders on the primary outcome variables, which were allergic symptoms and atopy (as shown in Table 5). The study found  keeping animals in the house increased the odds of atopy by 3.72 times (OR 3.72 [95% CI: 1.06, 13.1], *p* =0 .041). Morover, parental history of allergy was significantly associated with the development of allergic symptoms in the children witha paternal history of allergy (OR 4.15 [95% CI: 2.10, 8.23], *p* = 0.0001). Similarly, maternal history of allergy (OR 3.25 [95% CI: 1.63, 6.48], *p* = 0.001) was also associated with increased risk. Interestingly, the study also revealed that using charcoal as a source of fuel everyday was associated with a decreased risk of developing allergic symptoms (OR 0.5 [95% CI: 0.30, 0.84], *p* = 0.009).

**Table 5:** Potential risk factors for allergy and skin sensitization, Batu, Ethiopia 2016.

Variables	*n* (%)	Any sensitization	*p*	Any allergy symptoms	*p*
Maternal education (formal vs. **none**)	267 (57.9%)	1.27 (0.37, 4.39)	0.709	1.29 (0.81, 2.10)	.287
Maternal allergic history (yes vs. **no)**	38 (8.3%)	1.09 (0.14, 8.77)	0.93	**3.25** (**1.63, 6.48)**	**.001** a
Paternal allergic history (yes vs. **no**)	38 (8.3%)	2.5 (0.52, 12.01)	0.253	**4.15** (**2.10, 8.23)**	**<.0001** a
Breast fed till age 3 years (not fed vs. **Fed**)	262 (64.5%)	0.56 (0.39,5.70)	0.562	1.24 (0.73, 2.09)	.429
Older siblings					
0	174 (38.2%)	1.23 (0.14, 10.79)	0.853	1.9 (0.75, 4.82)	.176
1–3	238 (52.3%)	0.90 (0.10, 7.86)	0.92	1.36 (0.51, 3.43)	.514
4–10	43 (9.5%)	1		1	
De‐worming medication (yes vs. **no**)	331 (72.6%)	1.01 (0.27, 3.90	0.979	0.91 (0.55, 1.52)	.726
Proper latrine					
None, bush, field	6 (1.3%)	N/A	N/A	1	
Traditional pit	430 (93.5%)	N/A	N/A	3.00 (0.30, 29.94)	.349
Flush toilet	24 (5.2%)	N/A	N/A	1.21 (0.14, 10.53)	.86
Animals kept in the house (yes vs. **no**)	63 (13.7%)	**3.72** (**1.06, 13.1)**	**0.041** a	1.26 (0.67, 2.37)	.469
Smoking in the residential (yes vs. **no**)	20 (4.4%)	N/A	N/A	1.37 (0.48, 3.87)	.554
Charcoal fuel use					
Never	95 (20.6%)	1		1	
Sometimes	50 (10.8%)	1.23 (0.21, 7.90)	0.793	0.43 (0.18, 1.04)	.061
Everyday	316 (68.5%)	0.59 (0.14, 2.39)	0.459	**0.50** (**0.30, 0.84)**	**.009** a
Insecticide use (yes vs. **no**)	145 (31.5%)	1.22 (0.36, 4.39)	0.713	1.47 (0.92, 2.36)	.11
Vaccination (yes vs. **no**)	451 (97.8%)	N/A	N/A	0.43 (0.05, 3.42)	.428
Any protozoan infection (no vs. **yes**)	334 (75.2%)	0.36 (0.04, 2.60)	0.289	1.07 (0.63, 1.81)	.796
Atopy (yes vs. **no**)	11 (2.4%)	N/A	N/A	3.32 (0.99, 11.1)	.052

*Note*: Bold group taken as referent groups. 0 = Available number too small to calculate estimate.

Abbreviation: N/A, not available.

^a^ Significant association at *a* = .05 level.

## 4 DISCUSSION

In our study of children, we found that there is a weak negative correlation between helminth infection and atopy/allergy, although it did not reach statstical significance. Nevertheless, we did find that having animals in the house is significantly associated with atopy and history of allergy disorders in either the mother or father was significantly associated with the children's allergic symptoms.

The prevalence of allergic conditions reported in this study was similar to previous studies conducted in different parts of Ethiopia, except for eczema. In this study prevalence of various self‐reported allergic conditions were as follows: asthma, 2.2%; eczema, 13.2%; and hay fever, 6.9%. Other studies carried out in Gondar, Addis Ababa, and Jimma showed a prevalence of asthma 2.2%, 2.8%, and 2.2%; eczema 2.7%, 11.2%, and 5.6%; and hay fever 5.1%, 7.5%, and 4.5%, respectively. In addition, the magnitude of the atopic dermatitis (eczema) was found to be 9.6% in a research carried out in Mekelle. We couldn't fin an&amp;#x000A0;obvious reason to explain the relatively elevated frequency of eczema in our study. However, some unidentified environmental and socioeconomic factors may have played a role.^16^, ^17^, ^18^, ^19^

This study shows skin sensitization to *D. pteronyssinus* and cockroach (1.1% and 1.5%, respectively) was lower when compared with a study in Butajira (10.8% and 8.2%, respectively).^20^ These could possibly be explained by the differences in location.^21^

We also found a lower burden of helminth infections (11.9%) in our study compared to studies in southwest Ethiopia (43.7%), Babile (27.2%) and Gamo area (24.2%).^22^, ^23^, ^24^ Possible explanations for this noticeable difference could be the deworming program that is recently launched in the country and provided to our study population, differences in geographic locations, latrine coverage, and coverage of prevention control programs.

Our result demonstrated a borderline significant correlation between a positive Skin Prick Test and the occurance of any allergy symptom. Individuals who tested positive in the Skin Prick Test were 3.32 times more likely to develop one of the three allergic conditions (asthma, eczema, or hay fever). This agrees well with a study in Gondar^16^ and Butajira.^21^

This study found no significant link between helminth and atopy/allergy. This is consistent with findings presented by Amberbir et al.^25^ and Davey et al.^21^ that reported no association. However, our results go against Scrivener et al in Gondar, which reported an association of hookworm infection with a reduced risk of wheeze.^16^ Another study by Webb et al.,^26^ conducted in Uganda, provided strong evidence that individuals with certain helminths were more prone to atopy, also contradicting our findings. Calvert et al.,^27^ in South Africa, reported Ascaris infection was associated with a decreased risk of a positive skin test response but an increased risk of exercise‐induced bronchospasm. Variations in the study designs, the different types of helminths and also the intensity of infection could explain the differences in associations.

In this study, it was observed that the median total IgE was higher in the group “only helminth‐infected” and “both allergic and helminth‐infected”. However, the overall difference among all four groups was not statstically significant. This rise is expected because both helminths and allergic conditions are both associated with elevated levels of total IgE. A similar nonsignificant difference among the groups was reported in Gondar.^28^ The median total IgE (437 IU/mL) in “both allergic and helminthic” groups in this study's result was somehow close to those found in a Brazilian study (660 IU/mL)^29^ but different from a report from a study in Gondar (1411 kU/L).^28^

When compared with other studies the overall mean total IgE concentration of this study population was quite low (418 IU/mL). A study in Wondo Genet area, Ethiopia showed “only helminth‐infected” groups to have around a mean total IgE concentration of 1400^30^ and 1044 IU/mL in Ethio‐Israeli groups^31^ dissimilar to our result. Possible reasons for the general lower total IgE concentration could be due to the low prevalence of both helminths and allergic conditions, which have proved to elevate total IgE. A low total IgE levels was reported by Dydak et al.^32^ that concluded exposure of farm animals currently and in first year of life may result in a lower level of total IgE. The study design difference could have also impacted the result. Additionally, the low egg burden of the helminth infections (heavy infections are associated with high IgE) could also be a possible reason.

A similar, nonsignificant total IgE difference was found among the four helminth/atopy groups. Relatively lower median total IgE was observed in the “both helminth and atopy group” could be explained by the small number of participants that fall in that group.

Eosinophil distribution among the groups also showed no significant difference. As expected the helminth/atopy group showed elevated amount of eosinophil when compared to the other groups, as atopy and helminth infections are both associated elevated number of eosinophils.

This study supports that both genetics and environmental factor affect the development of allergic conditions or skin sensitization. Parental history of allergy was shown to increase allergic symptoms in the children was shown to increases skin sensitization in our finding. A study by Amberbir et al.^25^ agrees that parental history of wheeze increase the risk of allergy for the child. Keeping animals in the house was shown to increase skin sensitization in our study which goes against a study by Platts‐Mills et al.^33^ This difference could be due to their focus on only cat's allergen while we searched for any kind of animals.

Our result showed using charcoal everyday as a source of fuel decreases the risk of developing allergic symptoms which agrees well with Tilp et al.^34^ Their study showed cigarette smoke even without nicotine can reduce allergic Th2 responses. This topic remains controversial.

In conclusion, even though our study shows a nonsignificant association between helminth and atopy/allergy, the number of helminth‐infected participants have a noticeable low atopic and allergic conditions, indicating an inverse association. We recommend a high‐powered longitudinal study to rule out the possibility of masked association between helminth infection and allergic disorders. Immunological studies integrated with implementation of helminth control measures may elucidate how helminth elimination contributes to ongoing epidemics of inflammatory diseases.

## AUTHOR CONTRIBUTIONS

Bineyam Taye conceived and designed the study, supervised, collected data in the field and critically reviewed the manuscript. Sosina Walelign collected data, performed data analysis and interpretation, and prepared the manuscript. Geremew Tasew contributed essential reagents/tools and critically reviewed the manuscript. Mheret Tesfaye participated in data collection and interpretation, performed analysis, and critically reviewed the manuscript. Aster Tsegaye and Kassu Desta participated in data collection and interpretation and critically reviewed the manuscript. All authors read and approved the final manuscript.

## CONFLICT OF INTEREST STATEMENT

The authors declare no conflict of interest.

## Supporting information

Supporting Information

## Data Availability

Not applicable.
