# Supplementary material for: Association between helminth infection and allergic disorders among children in Batu, Ethiopia
Source: Immun Inflamm Dis. 2024 Mar 22;12(3):e1222. doi: 10.1002/iid3.1222 (PMC10959016; doi:10.1002/iid3.1222)
Supplement: Supplementary file 1 — Supporting Information [file IID3-12-e1222-s001.docx]

**Association of Intestinal Helminth Infections with Atopy and Allergic Symptoms in Young Children in Batu, Ethiopia: A cross sectional study**

**Helminth Infections and Atopy & Allergy Symptoms**

Sosina Walelign^1^, Mheret Tesfaye^2^, Geremew Tasew^3^, Kassu Desta^1^, Aster Tsegaye^1^, Bineyam Taye^4^

1 Department of Medical Laboratory Sciences, Addis Ababa University, Addis Ababa, Ethiopia

2 Bacteriology and Mycology National Reference Laboratory, Ethiopian Public Health Institute, Addis Ababa, Ethiopia

3 Director for Bacterial, Parasitic, and Zoonotic Diseases Research Directorate, Ethiopian Public Health Institute, Addis Ababa, Ethiopia

4 Department of Biology, Colgate University, New York, USA

Corresponding author: Sosina Walelign^1^, Department of Medical Laboratory Sciences, College of Health Sciences, Addis Ababa University, Addis Ababa, Ethiopia.

Telephone number: +251913899173

E-mail: [arkisosi@gmail.com](mailto:arkisosi@gmail.com) or [Sosina.walelign@aau.edu.et](mailto:Sosina.walelign@aau.edu.et)

Authors Contributions: BT conceived and designed the study, supervised and collected data in the field. SW Collected data, performed data analysis and interpretation, prepared the manuscript. GT contributed essential reagents/tools and critically reviewed the manuscript. MT participated in data collection and interpretation, performed analysis and critically reviewed the manuscript. AT, KD participated in data collection and interpretation and critically reviewed the manuscript.

All authors read and approved the final manuscript.

**ABSTRACT**

**Background** Helminths are potent immunomodulators and in their chronic infection state they may protect against allergy-related disease and atopy. On the other hand, they are also known for inducing allergic conditions. This study was aimed to assess the association between helminths and atopy and allergic conditions.

***Methods*** A total of 461 school children participated in this cross-sectional study. Data on allergic symptoms and a range of confounding variables was gathered from parents via an interviewer-led questionnaire. Skin sensitization to house dust mite and cockroach was analysed and a stool sample was collected for helminth analysis. Serum total Immunoglobulin E using ELISA and eosinophil count were also measured.

***Results*** Overall sensitivity to both allergens was 2.4%. Self - reported allergic outcomes in the last 12 months for the 461 participants had been; wheeze 3.7%, asthma 2.2%, eczema 13.2%, and hay fever 6.9%. A burden of 11.9% (53/444) was recorded for helminths. A borderline significant association was found between atopy and any allergy symptoms [OR 3.32 (95% CI: 0.99, 11.1), P = 0.052]. There was no significant association between helminths and atopy [OR 0.64 (95% CI: 0.29, 1.41) p = 0.268] and also between helminths and allergic symptoms [OR 0.64 (95% CI: 0.29, 1.41) p = 0.268]. Bivariate analysis showed keeping an animal in the house increases the risk of atopy while maternal and paternal history of allergy increases the risk of developing allergic symptoms in the children.

***Conclusion and Clinical Relevance*:** A non-significant association between helminths and atopy/ allergic disordered was found in this study, likely due to reduced statistical power, resulted in a lower prevalence of atopic and allergic conditions. Even though the analysis shows a non-significant association the number of helminth infected participants have a noticeable low atopic and allergic conditions indicating an inverse association. Further high-powered longitudinal studies are necessary to clarify the controversy further and lead to potential alternative drug options for allergies.

**Keywords:** Atopy, helminths, Allergy, IgE

**INTRODUCTION**

**Background**

Helminth infections and allergy disorders are common causes of morbidity around the globe though their distribution shows an inverse association [1-3]. A potential role of parasitic infections having a protective role against autoimmune disorders by parasite‐mediated immune suppression was implicated in 1960s by greenwood [4].

The human immune response to a geohelminth infection and allergic disease has a close resemblance. Helminth infections have been associated with regulatory state that impairs both responses to parasite specific and bystander antigens. Some of immune regulatory mechanisms helminths use are Treg cells expansion [5], enhanced production of IL-10 and TGF-ß [6], M2 macrophages [7] and the list continues.

The interest that rose in 1960’s to investigate the effect of helminths on allergy still goes on today without reaching a consensus on their protective or inducing role. Regardless of the conflicting results from studies, the current science has shown advancement as far as working on trials that aim to treat immune-mediated diseases using helminth-derived molecules. Such helminth-derived molecules could be used to selectively mimic the desirable regulatory effects of parasitic helminths, without the side-effects of infection, in new therapeutic approaches for immune-mediated diseases [8,9].

Low income countries are highly burdened with intestinal helminth infections [10] making them an ideal place to conduct studies on interaction between atopic disorders and intestinal helminth infections. On the other hand, there is an increase in allergy [3] in some urban areas of developing countries which might be linked with the improved treatment in urban than rural areas. Hence understanding the relationship between helminths and allergy could gear the future treatment strategies and provide an insight about risks and benefits of eradicating helminth infections in endemic areas. Therefore, we studied the association between helminth infections and atopy or allergic diseases in Ethiopian context.

**Objectives**

## General objective

To assess the association of intestinal helminth infection with atopy and allergic outcomes in young children in Batu, Ethiopia.

**Specific objectives**

- To determine the association between intestinal helminths with atopy
- To determine the association between intestinal helminths with allergic outcomes
- To compare serum total IgE and peripheral eosinophil counts among different groups of study participants
- To assess the association between atopy and allergy
- To determine the associated risk factors for atopy and allergic outcomes

MATERIALS AND METHODS

## Study design

A Hospital and school based cross sectional study was conducted.

# Study area

The research project was done at Batu (Ziway), Ethiopia. The town is located in Oromia National Regional State, in East Shoa zone, Adami Tulu Jiddo Woreda, at a distance of 160 Km from Addis Ababa. Its astronomical location is 7º 56’ North Latitude and 38º 43’ East Longitude with an elevation of 1643 meters above sea level. Batu town was founded in 1961 [25]. Adjacent to [Lake Ziway](https://en.wikipedia.org/wiki/Lake_Ziway) (Lake Dambal), the economy of the town is based on [fishing](https://en.wikipedia.org/wiki/Fishing) and [horticulture](https://en.wikipedia.org/wiki/Horticulture) [26]. Study participants were recruited from five sites; Batu Hospital, Sher Hospital, Batu 1 Health Center, Sher Elementary School and Batu Elementary School.

## Study period

The study was conducted from October 2015 – May 2017. Actual data collection period was from May-June 2016.

## Population

### Source population

Young children visiting the selected facilities during the study period were the source population.

### Study population

Young children (2-14 years of age) who were present at the five sites during the study period and who qualified our inclusion criteria included in the study.

## Eligibility

### Inclusion criteria

 Young children (2-14) years who visited the five sites during the study period whose parents volunteered to take part.

### Exclusion criteria

- Children who received anti- helminthic drugs for the last one month
- Children who received drugs that interfere with the skin prick test response
  - Second generation antihistamines
  - Antidepressants such as doxepin, other tricyclics, and tetracyclics have antihistamine activity and may need to be withheld for 1-2 weeks or more.
  - Phenothiazines also have antihistamine activity
  - Over the counter cold and flu remedies, “sinus” analgesics, antitussives; antiemetics, sedatives, relaxants, migraine prophylactics (cyproheptadine, pizotifen).
  - Prolonged topical corticosteroids have been shown to reduce skin reactivity

## Study variables

### Dependent variables

- Atopic status (SPT) and allergy symptoms
- Total IgE profile
- Eosinophil count level

### Independent variable

- Socio demographic characteristics
- Intestinal helminth infection
- Other associated risk factors
  - Maternal education
  - Maternal allergic history
  - Paternal allergic history
  - Breast fed till age 3
  - Number of older siblings  De-worming medication
  - Proper latrine
  - Animals kept in the house
  - Presence of smokers in the house
  - Charcoal fuel use
  - Insecticide use
  - Vaccination history

### Sampling technique

Convenient sampling technique was used.

# Operational Definitions

- **Atopy**: A skin prick test result with a wheal size of 3mm and above for the house dust mite (*Dermatophagoides pteronyssinus)* and German cockroach (*Blattella germanica*) allergens.
- **Allergic participants**: Participants who report one of the following; wheeze, asthma, eczema or hay fever within the last 12 months.
- **Formal education vs. Illiterate**: Those who can read and write versus those who cannot read and write.
- **Helminth infected**: Any helminth infection detected either by direct wet mount or formol ether concentration technique.
- **Young children:** refers to children aged 2-14 years

## Sample size calculation and sampling technique

### Sample size calculation

The minimum required sample size to determine a single population proportion was calculated using the following formula.


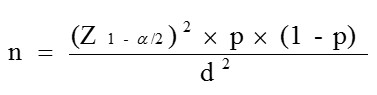


Where,

n= the sample size estimate, P= 50%, Za/_2_ is 1.96 n= (1.96)^2^(0.05)(0.05)

(0.0225)^2^

n= 384

A contingency of 38.4 (422) participants was planned to be added. We enrolled a total of 461 participants surpassing the contingency plan.

# RESULTS

## Demographic and socioeconomic characteristics of Study participants

A total of 461 eligible study participants were recruited from five sites: Batu Hospital (3, 0.7%), Sher

Hospital (103, 22.3%), Batu 1 Health Center (7, 1.5%), Sher Elementary School (160, 34.7%) and Batu Elementary School (188, 40.8%). Just over half were female (50.8%) and majority (96.1%) were from urban areas. The mean age was 8.8 years (the age range being between 2 and 14) for those 458 participants. Majority of the participants (97.9%) had a proper (any) vaccination. 57.9% of the participants’ mothers had a formal education and 43.8% were housewives. Table 1 describes the detailed characteristics of the study participants.

**Table1:** Socio demographic characteristics of the study participants, Batu, Ethiopia 2016.

| **Variables** |  | **Number** | **Percent** |
| --- | --- | --- | --- |
| **Sex (N=461)** | Male | 227 | 49.2 |
|  | Female | 234 | 50.8 |
| **Age* (N=458)** | 2-4 | 37 | 8.1 |
|  | 5-9 | 229 | 50 |
|  | 10-14 | 192 | 41.9 |
| **Residency (N=461)** | Rural | 18 | 3.9 |
|  | Urban | 443 | 96.1 |
| **Maternal education(N=461)** | Formal | 267 | 57.9 |
|  | Cannot read and write | 194 | 42.1 |
| **Maternal occupation (N=461)** | Farming and related | 88 | 19.1 |
|  | Trading and related | 73 | 15.8 |
|  | Government employee | 12 | 2.6 |
|  | Housewife | 202 | 43.8 |
|  | Others | 86 | 18.7 |
| **Vaccination history (N=461)** | Never vaccinated | 10 | 2.2 |
|  | Vaccinated | 451 | 97.8 |

*Age classified based on “Ethiopia Mini Demographic and Health Survey 2014”. Central Statistical Agency. Addis Ababa, Ethiopia. 2014.

## Allergic sensitization and self - reported allergic symptoms

Skin Prick Test was performed for 454 participants for the two allergens, house dust mites (*Dermatophagoides pteronyssinus)* and German cockroach (*Blattella germanica*), with rate of 1.1% and 1.5 % sensitivity, respectively. Overall sensitivity or atopic status was 2.4 %. Only one participant was sensitive to both allergens. Self - reported allergic outcomes in the last 12 months for the 461 participants had been; wheeze (3.7%), asthma (2.2%), eczema (13.2%), and hay fever (6.9%). Only 5 participant’s asthmatic status was confirmed by a doctor. Table 2 describes the frequency of each type of self - reported allergic symptoms and allergic sensitization.

**Table 2:** Frequency of allergic sensitizations and self - reported allergic symptoms of young children, Batu, Ethiopia 2016.

| **Variables** | **Number** | **Percent** |
| --- | --- | --- |
| **Self - reported allergic symptoms (N= 461)** |  |  |
| Wheeze | 17 | 3.7 |
| Asthma | 10 | 2.2 |
| Hay fever | 32 | 6.9 |
| Eczema | 61 | 13.2 |
| Any allergy symptom | 94 | 20.4 |
| **Skin Prick Test (N= 454)** |  |  |
| HDM * (*Dermatophagoides pteronyssinus)* | 5 | 1.1 |
| German cockroach (*Blattella germanica*) | 7 | 1.5 |
| Sensitization to both allergens | 1 | 0.22 |
| Any sensitization ^§^ | 11 | 2.4 |

Both atopic and allergic 5 1.1

*HDM – house dust mite

**§** Sensitization either *to Dermatophagoides pteronyssinus* or German cockroach (*Blattella germanica)*

## Burden of Helminth parasites

Stool sample collected from the participants was tested with direct microscopic examination (Wet mount) and Formol-Ether concentration techniques. Merged results from the two tests were used for the analysis. An 11.9% (53/444) burden of helminths was recorded. *Hymenolepis nana* was found to be the most frequent 31(58%) helminth. *Ascaris lumbricoides 2* (4%), Hookworm species 3(6%), *Schistosoma mansoni 3* (6%), *Trichuris trichuria* 1 (2%), Tenia species 3 (6%), *Enterobius vermicularis* 7 (13%), mixed helminth infection 3 (5%) * were frequencies of the helminths. Based on egg load determination, all infections were light infections. The maximum number of eggs reported was 22 eggs/ gram of faeces. Based on egg load determination, all infections were light infections. The maximum number of eggs reported was 22 eggs/ gram of faeces. Feces

*Mixed infection: (*Trichuris trichuria/ Schistosoma mansoni, Schistosoma mansoni/ Schistosoma mansoni and Enterobius vermicularis/ Hymenolepis nana*)

## Association of helminthes and protozoa infection with allergic symptoms and Atopy

Overall helminth and protozoa infections were used to see the association with allergic symptoms. Individual helminth was not used for analysis since the prevalence of each helminth was small to yield an appropriate valid association. Neither helminth [OR 0.64 (95% CI: 0.29, 1.41) p = 0.268] nor protozoa [OR 0.93 (0.56, 1.58), P=0.796] were significantly associated with allergy symptoms (Table 3).

**Table 3.** Associations between allergic conditions and intestinal parasites infection among young children, Batu, Ethiopia, 2016.

|  |  | **Any allergic conditions** | | |  |
| --- | --- | --- | --- | --- | --- |
| **Variables** | **Overall**  **N** | **Yes n(%)** | **No n(%)** | **Crude OR (95 % CI)** | **P-value** |
| **Helminthes**  No  Yes |  |  |  |  |  |
|  | 391  53 | 85(21.7%)  8 (15.1%) | 306(78.3%)  45(84.9%) | 0.64 (0.29, 1.41) 1 | 0.268 |
| **Protozoa**  No  Yes |  |  |  |  |  |
|  | 334  110 | 69(20.7%)  24(21.8%) | 265(79.3%)  86(78.2) | 0.93 (0.56, 1.58) 1 | 0.796 |

Though not being infected with protozoa seemed to increase the risk of atopy by 3.08 times, as Table 4 shows the association was not statistically significant. The association between helminth infections and atopy was not statistically significant as well. Even though the analysis shows a non-significant association the number of helminth infected participants have a noticeable low atopic and allergic conditions indicating an inverse association.

**Table 4.** Associations between atopy and helminth & protozoan infection among school children of five selected facilities, Batu, Ethiopia, 2016.

|  |  |  | **Atopy** |  |  |  |
| --- | --- | --- | --- | --- | --- | --- |
| **Variables** | **Overall**  **N** | **Yes n(%)** |  | **No n(%)** | **Crude OR (95 % CI)** | **P-value** |
| **Helminthes** |  |  |  |  |  |  |

51 49(96.1%)

No

Yes

387

8(2.1%)

379 (97.9%)

0.52(0.11, 2.51)

1

0.413

2(3.9%)

**Protozoa**

No

328

9(2.7%)

319 (97.3%)

3.08(0.39, 24.55)

1

0.289

Yes

110

(0.9%

)

1

109

(99.1%)

## Distribution of total IgE and Eosinophils among the different groups of the study participants

Independent sample Kruskal Wallis test was used to check if there was a significant mean rank difference of total IgE concentrations distribution among the different helminth/atopy (Figure 5) and helminth/allergy (Figure 6) groups of the study participants. The analysis showed the distribution of total IgE did not differ significantly across the categories of both helminth/allergy (p=0.136) and helminth/atopy (p=0.147) groups. As shown in Figure 6, there is an increasing pattern in the total IgE level from no helminthes no atopy to those with both helminthes and atopy groups.


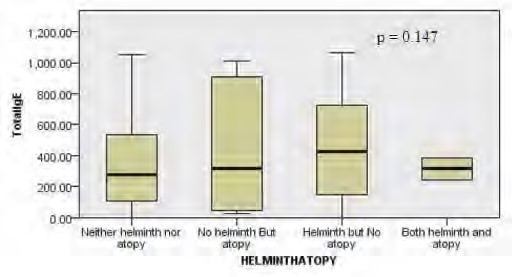


n=362 n=8 n=47

n=2

**Figure 5:** Distribution of total IgE among the different helminth/atopy groups Batu, Ethiopia, 2016.


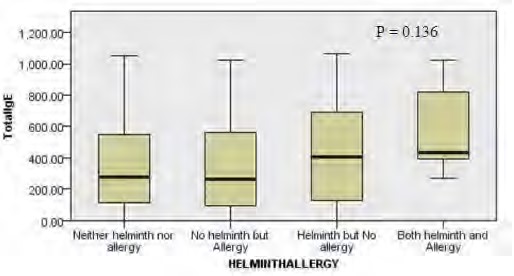


n=295 n=79

n=43

n=8

**Figure 6:** Distribution of total IgE among the different helminth/allergy groups Batu, Ethiopia, 2016.

Since there were outliers in eosinophil distribution, independent sample median test was used to check if there was a significant median difference of among the different helminth/allergy (Figure 7) and helminth/atopy groups (Figure 8). Both revealed a non-significant association.


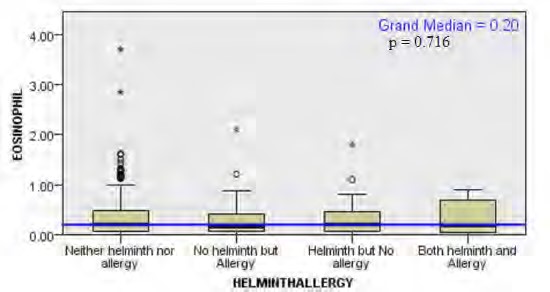


**Figure 7:** Distribution of Eosinophil among the different helminth/allergy groups Batu, Ethiopia, 2016.


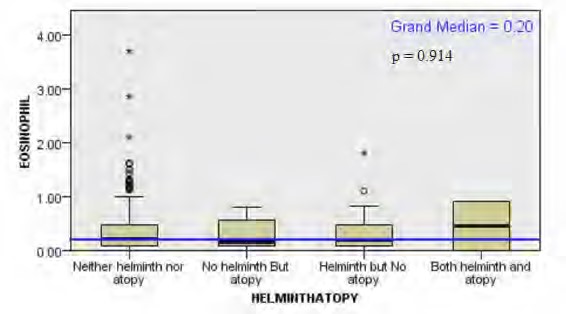


**Figure 8:** Distribution of Eosinophil among the different helminth/atopy groups Batu, Ethiopia, 2016.

**Association between skin sensitization (atopy) and allergy symptoms**

A borderline significant association was found between atopy and any allergy symptoms. Individuals with a positive SPT response to any allergen were 3.32 times more likely to report any allergy symptom [OR 3.32 (95% CI: 0.99, 11.1), P = 0.052]. Table 5 describes the association between atopy and allergy symptoms. **Table 5:** Association between atopy and allergy symptoms, Batu, Ethiopia 2016.

**Potential risk factors for allergy and skin sensitization**

Different factors were observed to be associated with having allergic symptoms and atopy (Table 7). Bivariate analysis showed keeping animals in the house increased the odds of atopy by 3.72 times [OR 3.72, (95% CI: 1.06, 13.1) p = 0.041]. In addition, parental history of allergy was significantly associated with developing allergic symptoms in the children; Paternal history of allergy [OR 4.15 (95% CI: 2.10, 8.23) p = 0.000] was shown to increase the risk of developing allergic symptoms, similarly maternal history of allergy [OR 3.25 (95% CI: 1.63, 6.48) p = 0.001] was associated with increased risk. Our result showed using charcoal everyday as a source of fuel is associated with decreased risk of developing allergic symptoms [OR 0.5 (95% CI: 0.30, 0.84) p = 0.009].

**Table 6:** Potential risk factors for allergy and skin sensitization in young children, Batu, Ethiopia 2016.

| **Variables** | **n%** | **Any sensitization** | **P-value** |  | **Any allergy symptoms** | **P-value** |
| --- | --- | --- | --- | --- | --- | --- |
| Maternal education (formal vs. **none**) | 267 (57.9%) | 1.27 (0.37, 4.39) | 0.709 |  | 1.29 (0.81, 2.10) | 0.287 |
| Maternal allergic history (yes vs **no)** | 38 (8.3%) | 1.09 (0.14, 8.77) | 0.93 |  | **3.25 (1.63, 6.48)** | **0.001*** |
| Paternal allergic history (yes vs **no**) | 38 (8.3%) | 2.5 (0.52, 12.01) | 0.253 |  | **4.15 (2.10, 8.23)** | **0*** |
| Breast fed till age 3 (**Fed** vs not fed) | 262 (64.5%) | 0.56 (0.39,5.70) | 0.562 |  | 1.24 (0.73, 2.09) | 0.429 |
| Older siblings |  |  |  |  |  |  |
| 0 | 174 (38.2%) | 1.23 (0.14, 10.79) | 0.853 |  | 1.9 (0.75, 4.82) | 0.176 |
| 1 to 3 | 238 (52.3%) | 0.90 (0.10, 7.86) | 0.92 |  | 1.36 (0.51, 3.43) | 0.514 |
| 4 to 10 | 43 (9.5%) | 1 |  |  | 1 |  |
| De-worming medication (yes vs **no**) | 331 (72.6%) | 1.01 (0.27, 3.90 | 0.979 |  | 0.91 (0.55, 1.52) | 0.726 |
| Proper latrine |  |  |  |  |  |  |
| None, bush, field | 6 (1.3%) | 0 | 0 |  | 1 |  |
| Traditional pit | 430 (93.5%) | 0 | 0 |  | 3.00 (0.30, 29.94) | 0.349 |
| Flush toilet | 24 (5.2%) | 0 | 0 |  | 1.21 (0.14, 10.53) | 0.86 |
| Animals kept in the house (**no** vs yes) | 63 (13.7%) | **3.72 (1.06, 13.1)** | **0.041*** |  | 1.26 (0.67, 2.37) | 0.469 |
| Smoking in the residential (**no** vs yes) | 20 (4.4%) | 0 | 0 |  | 1.37 (0.48, 3.87) | 0.554 |
| Charcoal fuel use |  |  |  |  |  |  |
| Never | 95 (20.6%) | 1 |  |  | 1 |  |
| Sometimes | 50 (10.8%) | 1.23 (0.21, 7.90) | 0.793 |  | 0.43 (0.18, 1.04) | 0.061 |
| Everyday | 316 (68.5%) | 0.59 (0.14, 2.39) | 0.459 |  | **0.50 (0.30, 0.84)** | **0.009*** |
| Insecticide use (yes vs **no**) | 145 (31.5%) | 1.22 (0.36, 4.39) | 0.713 |  | 1.47 (0.92, 2.36) | 0.11 |
| Vaccination (yes vs **no**) | 451(97.8%) | 0 | 0 |  | 0.43 (0.05, 3.42) | 0.428 |
| Any protozoan infection (**yes** vs no) | 334(75.2%) | 0.36 (0.04, 2.60) | 0.289 |  | 1.07 (0.63, 1.81) | 0.796 |
| Atopy (yes vs **no**) | 11 (2.4%) | 0 |  | 0 | 3.32 (0.99, 11.1) | 0.052 |

***significant association at a=0.05 level**

**Bold group – taken as referent groups**

**0 = Available number too small to calculate estimate.**

# DISCUSSION

In this cross sectional study of young children, association between helminth and atopy/allergy and also the distribution of total IgE was assessed. The result showed that there was no significant association between helminths and atopy and also between helminths and allergy. Bivariate analysis showed keeping animals in the house associated with atopy while maternal and paternal history of allergy associated with allergic symptoms in the children.

Since there was no previous similar study or survey done in this particular study area it was difficult to estimate the burden of both atopic status and allergic conditions of this particular study population. In the end, this cross sectional study showed the prevalence of allergic conditions were comparable to previous studies from different parts of Ethiopia except with eczema. Prevalence of various selfreported allergic conditions were; asthma 2.2%, eczema 13.2%, and hay fever 6.9%. Other studies carried out in Gondar, Addis Ababa, and Jimma showed a prevalence of asthma 2.2%, 2.8%, and 2.2%, eczema 2.7%, 11.2%, and 5.6% and hay fever 5.1%, 7.5%, and 4.5%, respectively. In addition, the magnitude of the atopic dermatitis (eczema) was found to be 9.6 % in a research carried out in Mekelle. No obvious reason was found to explain the relatively elevated frequency of eczema in our study. However, some unidentified environmental and socioeconomic factors may have played a role [18, 32, 33, 34].

This study shows skin sensitization to *D. pteronyssinus* and cockroach (1.1% and 1.5% respectively) was low when compared with a study in Butajira (10.8% and 8.2% respectively) [35]. Or the differences in location can be a possible reason [36].

Like that of skin sensitization, the burden of helminths (11.9%) in our study was relatively low when compared to studies in southwest Ethiopia (43.7%), Babile (27.2 %) and Gamo area (24.2%) [37, 38, 39]. Possible explanation for this noticeable difference include the deworming program that is recently launched in the country and provided to our study population, differences in geographic locations, study population’s age, latrine coverage and coverage of prevention control programs. Different sensitivities of the techniques used for stool concentrations can provide a possible explanation.

Our result demonstrate a borderline significant association between positive Skin Prick Test and presentation of any allergy symptom. Individuals with a positive Skin Prick Test were 3.32 times more likely to develop one of the three allergic conditions (asthma, eczema or hay fever). This agrees well with a study in Gondar [18] and Butajira [35].

Results of this study revealed no significant association between helminth and atopy/allergy. This is consistent with findings presented by Amberbir *et al* that reported no association [22] and Davey *et al.,* [35]. However, our results go against Scrivener *et al* in Gondar, which reported an association of hookworm infection with a reduced risk of wheeze [18]. Another study by Webb *et al.,* conducted in Uganda provided strong evidence that individuals with certain helminths were more prone to atopy, also contradicting our findings [20]. Calvert *et al.,* in South Africa reported Ascaris infection was associated with a decreased risk of a positive skin test response but an increased risk of exercise-induced bronchospasm [21]. Possible explanations for the difference in associations could be from variations in the study designs, the different types of helminths and also the intensity of infection.

In addition to assessing the association between helminths and allergic condition, this study tried to determine the total IgE concentration in this study population. The result showed, the median total IgE concentration in the “only helminth infected” and “both allergic and helminth infected” groups of our study were found to be elevated even though the overall difference among the four groups was not statistically significant. This elevation is expected since helminths and allergic conditions are both associated with elevated levels of total IgE. A similar non-significant difference among the groups was reported in Gondar [41]. The median total IgE (437 IU/ml) in “both allergic and helminthic” groups in this study’s result was somehow close to those found in a Brazilian study (660 IU/ml) [40] but different from a report from a study in Gondar (1411 kU/L) [41].

When compared with other studies the overall mean total IgE concentration of this study population was quite low (418 IU/ml). A study in Wondo Genet area, Ethiopia showed “only helminth infected” groups to have around a mean total IgE concentration of 1400 IU/ml [42] and 1044 IU/ml in EthioIsraeli groups [43] dissimilar to our result.

Possible reasons for the general lower total IgE concentration could be due to the low prevalence of both helminths and allergic conditions, which have proved to elevate total IgE. The cross-sectional method used in this study instead of a case control studies could have also impacted the result.

Additionally, the low egg burden of the helminth infections (heavy infections are associated with high IgE) could also be a possible reason.

A similar, non-significant total IgE difference was found among the four helminth/atopy groups. Relatively lower median total IgE was observed in the “both helminth and atopy group” could be explained by the small number of participants that fall in that group.

Eosinophil distribution among the groups also showed no significant difference. As expected the helminth/atopy group showed elevated amount of eosinophil when compared to the other groups since atopy and helminth infections are both associated elevated number of eosinophils.

This study supports that both genetics and environmental fact or affect the development of allergic conditions or skin sensitization. Parental history of allergy was shown to increase allergic symptoms in the children was shown to increases skin sensitization in our finding. A study by Amberbir *et al,* agrees that parental history of wheeze increase the risk of allergy for the child [22]. Keeping animals (Cats) in the house was shown to decrease skin sensitization in our study which goes against a study by PlattsMills *et al*., [44]. This difference could be due to their focus was only on cats allergen while we searched any kind of animals.

Our result showed using charcoal everyday as a source of fuel decreases the risk of developing allergic symptoms which agrees well with Tilp C *et al.,* [45]. Their paper showed cigarette smoke even without nicotine can reduce allergic Th2 responses. This topic remains controversial.

# Strength and limitation of the study

## Strength of the study

- Batu was a new site to explore this topic as to our knowledge (Previous studies were carried out in Addis Ababa, Jimma, Gondar)
- Provided a current information on Atopic and allergic status for the area

## Limitation of the study

- A cross sectional design was used
- Number of participants atopy and helminth infections were low in order to investigate their relationship
- Allergic symptoms are self - reported by the study participants and their guardians.
- We defined atopy with only two dominant aeroallergens, house dust mite and cockroach

# Conclusion and Recommendation

In conclusion, even though our study shows a non-significant association between helminth and atopy/allergy, the number of helminth infected participants have a noticeable low atopic and allergic conditions indicating an inverse association. We recommend a high-powered longitudinal study to rule out the possibility of masked association between helminth infection and allergic disorders. Immunological studies integrated with implementation of helminth control measures may elucidate how helminth elimination contributes to ongoing epidemics of inflammatory diseases.

**Funding**

- Funding Colgate University Research Council funded the study. The funding body had no role in study design; collection, analysis, and interpretation of data; writing of the report; or the decision to submit the paper for publication.

# 9. REFERENCES

1. Feary J, Britton J, Leonardi-Bee J. Atopy and current intestinal parasite infection: a systematic review and meta-analysis. *Allergy*. 2011; 66: 569–578.
2. Pullan RL, Smith JL, Jasrasaria R, Brooker SJ. Global numbers of infection and disease burden of soil transmitted helminth infections in 2010. *Parasites & Vectors*. 2014;7:37.
3. Weinberg EG. Urbanization and childhood asthma: an African perspective. *J Allergy Clin Immunol*. 2000;105:224–231.
4. Strachan DP. Hay fever, hygiene, and household size. *BMJ, 1989;* 229 (6710): 1259-1260.
5. Fallon PG, Mangan NE. Suppression of TH2 -type allergic reactions by helminth infection.

*Nature reviews*. 2007; 7: 220-230.

1. Cooper PJ, Chico ME, Sandoval C, Nutman TB. Atopic Phenotype Is an Important Determinant of Immunoglobulin E–Mediated Inflammation and Expression of T Helper Cell Type 2 Cytokines to Ascaris Antigens in Children Exposed to Ascariasis. *The Journal of Infectious Diseases*. 2004; 190:1338–46.
2. Carvalho EM, Bastos LS, Araújo MI. Worms and allergy. *Parasite Immunology.* 2006; 28: 525-534.
3. Fitzsimmons CM, Falcone FH, Dunne DW. Helminth allergens, parasite-specific IgE, and its protective role in human immunity. *Front.Immunol*. 2014;5:61.
4. Owen J, Punt J, Stranford S. *Kuby Immunology*. 7^th^ ed. New York. W. H. Freeman and Company. 2013.
5. Daniłowicz-Luebert E, O’Regan N, Steinfelder S, Hartmann S. Modulation of Specific and

Allergy-Related Immune Responses by Helminths. *Journal of Biomedicine and Biotechnology*.

2011.

1. Santiago HC, Ribeiro-Gomes FL, Bennuru S, Nutman T. Helminth infection alters IgE responses to allergens structurally related to parasite proteins. *J Immunol*. 2015; 194(1): 93– 100.
2. Dellen RJ, Thompson GH. Absence of intestinal parasites in asthma. *N. Engl. J. Med.* 1971; 285(3): 146–148
3. Erb KJ. Can helminths or helminth-derived products be used in humans to prevent or treat allergic diseases? *Trends in Immunology.* 2008; 30(2): 75-82.
4. Feary J, Britton J, Leonardi-Bee J. Atopy and current intestinal parasite infection: a systematic review and meta-analysis. *Allergy*. 2011; 66: 569–578.
5. Leonardi-Bee J, Pritchard D, Britton J, and the Parasites in Asthma Collaboration. Asthma and Current Intestinal Parasite Infection: Systematic Review and Meta-Analysis. Am J Respir Crit Care Med. 2006; 174: 514–523.
6. Flohr C, Tuyen LN, Lewis S, Quinnell R, Minh TT, Liem, HT et al. Poor sanitation and helminth infection protect against skin sensitization in Vietnamese children: A cross-sectional study. *J Allergy Clin Immunol*. 2006; 118(6): 1305-1311.
7. Stein M, Greenberg Z, Boaz M, Handzel ZT, Meshesha MK, Bentwich Z. The Role of Helminth Infection and Environment in the Development of Allergy: A Prospective Study of NewlyArrived Ethiopian Immigrants in Israel. *PLoS Negl Trop Dis*. 2016; 10(1).
8. Scrivener S, Yemaneberhan H, Zebenigus M, Tilahun D, Girma S, Ali S *et al*. Independent effects of intestinal parasite infection and domestic allergen exposure on risk of wheeze in Ethiopia: a nested case control study. *Lancet.* 2001; 358: 1493–99.
9. Buendía E, Zakzuk J, Mercado D, Alvarez A and Caraballo L. *World Allergy Organization Journal*. 2015; 8:8
10. Webb EL, Nampijja M, Kaweesa J, Kizindo R, Namutebi M, Nakazibwe E *et al.* Helminths are positively associated with atopy and wheeze in Ugandan fishing communities: results from a cross-sectional survey. *Allergy*. 2016; 71: 1156–1169.
11. Calvert J, Burney P. Ascaris, atopy, and exercise-induced bronchoconstriction in rural and urban South African children. *J* *Allergy Clin Immunol*. 2010; 125(1): 100–5
12. Amberbir A, Medhin G, Erku W, Alem R, Simms R, Robinson K, *et al.* Effects of Helicobacter pylori, geohelminth infection and selected commensal bacteria on the risk of allergic disease and sensitization in 3-year-old Ethiopian children. *Clinical & Experimental Allergy*. 2011; 41: 1422–1430.
13. Kitagaki K, Businga TR, RacilaD, Elliott DE, Weinstock JV, Kline JN. Intestinal Helminths Protect in a Murine Model of Asthma. *The Journal of Immunology*. 2006; 177: 1628 –1635
14. WHO. Soil-transmitted helminth infections. Fact sheet. 2016. <http://www.who.int/mediacentre/factsheets/fs366/en>. *(Accessed on January 25, 2016)*
15. [Zeway/Batu City Administration.](https://www.google.com.et/url?sa=t&rct=j&q=&esrc=s&source=web&cd=2&cad=rja&uact=8&ved=0ahUKEwiF2_f4xMnKAhXMHxoKHYe4CHYQFggfMAE&url=http%3A%2F%2Fwww.mwud.gov.et%2Fweb%2Fzeway%2Fhome&usg=AFQjCNFzPl4MKq5w3otc2sXQU9-KEdivMg&sig2=HZBTv7QBsDeze5YZBjb8vA&bvm=bv.112766941,d.bGQ) [www.mwud.gov.et/web/zeway/home](http://www.mwud.gov.et/web/zeway/home) *(Accessed on January 25, 2016)*
16. Ziway.  [https://en.wikipedia.org/wiki/Ziway*.*](https://en.wikipedia.org/wiki/Ziway) *(Accessed on January 25, 2016)*
17. ASCIA skin prick testing working party. Skin prick testing for the diagnosis of allergic disease.

A manual for practitioners. 2013.

1. Cheesbrough M. *District Laboratory Practice in Tropical Countries: Part 1.* Second Edition.

Cambridge. Cambridge University Press. 2009 www.cambridge.org/9780521676304.

(Accessed on December 1, 2015).

1. Total Human IgE ELISA kit. Diagnostic Automation/Cortez Diagnostics Inc. 2016.
2. Cheesbrough M. *District Laboratory Practice in Tropical Countries: Part 2.* Second Edition.

Cambridge. Cambridge University Press. 2009 www.cambridge.org/9780521676304.

(Accessed on December 1, 2015).

1. Mindray. BC-3000 Plus hematology analyzer manual. 2003.
2. Hailu S, Tessema T, Silverman M. Prevalence of Symptoms of Asthma and Allergies in School children in Gondar Town and Its Vicinity, Northwest Ethiopia. *Pediatr Pulmonol*. 2003;

35:427–432.

1. Melaku K. Atopic Eczema in School Children. *Ethiop J Health Dev*. 2000; 14(1): 105-108.
2. Kelbore AG, Alemu W, Shumye A, Getachew S. Magnitude and associated factors of Atopic dermatitis among children in Ayder referral hospital, Mekelle, Ethiopia. *BMC dermatology*. 2015;15:15.
3. Davey G, Vennw A, Belete H, Berhane Y, Britton J. Wheeze, allergic sensitization and geohelminth infection in Butajira, Ethiopia. Clin Exp Allergy 2005; 35:301–307.
4. Renz, H. TH1/TH2 immune response profiles differ between atopic children in eastern and western Germany. *J. Allergy Clin. Immunol.* 2002*;* **109**:338–342
5. Mengistu A, Gebre-Selassie S, Kassa T. Prevalence of intestinal parasitic infections among urban dwellers in southwest Ethiopia. *Ethiop.J.Health Dev*. 2007; 21(1):12-17.
6. Tadesse G. The prevalence of intestinal helminthic infections and associated risk factors among school children in Babile town, eastern Ethiopia. *Ethiop.J.Health Dev.* 2005; 19(2): 140-147.
7. Wegayehu T, Tsalla T, Seifu B, Teklu T. Prevalence of intestinal parasitic infections among highland and lowland dwellers in Gamo area, South Ethiopia. *BMC Public Health*. 2013 13:151.
8. Medeiros D, Silva AR, Rizzo JA, Motta ME, Oliveira FH, Sarinho ES. Total IgE level in respiratory allergy: study of patients at high risk for helminthic infection. *J Pediatr (Rio J*).

2006; 82:255–259

1. Selassie FG, Stevens RH, Cullinan P, Pritchard D, Jones M, Harris J, et al. Total and specific

IgE (house dust mite and intestinal helminths) in asthmatics and controls from Gondar, Ethiopia. *Clinical and Experimental Allergy*. 2000; 30: 356-358

1. Mulu A, Kassu A, Legesse M, Erko B, Nigussie D, Shimelis T *et al.* Helminths and malaria coinfections are associated with elevated serum IgE. *Parasites & Vectors*. 2014. 7:240
2. Kidon MI, Stein M, Geller-Bernstein C, Weisman Z, Steinberg S, Greenberg Z, *et al*. Serum

Immunoglobulin E Levels in Israeli- Ethiopian Children: Environment and Genetics. *Immunology and Allergies: IMAJ.* 2005;7: 799–802

1. Platts-Mills TA, Woodfolk JA, Erwin EA , Aalberse, R. Mechanisms of tolerance to inhalant allergens: the relevance of a modified TH2 response to allergens from domestic animals. *Springer Semin.Immunopathol*. 2004; 25: 271–279.
2. Tilp C, Bucher H, Haas H, Duechs MJ, Wex E, Erb KJ. Effects of conventional tobacco smoke and nicotine-free cigarette smoke on airway inflammation, airway remodeling and lung function in a triple allergen model of severe asthma. Clin Exp Allergy. 2016 Jul;46(7):957-72.
